# Minimally invasive trans-superior articular process percutaneous endoscopic lumbar discectomy with robot assistance

**DOI:** 10.1186/s12891-022-06060-8

**Published:** 2022-12-31

**Authors:** Zongjiang Wang, Ying Tan, Kai Fu, Zhaowu Meng, Liang Wang

**Affiliations:** 1 Department of Spinal Surgery, Sunshine Union Hospital, No. 9000, Yingqian street, Gaoxin District, 261041 Weifang City, Shandong Province China; 2 Department of Spinal Surgery, Weifang Traditional Chinese Medicine Hospital, 261041 Weifang, China

**Keywords:** Minimally invasive spinal surgery, Endoscopic spinal surgery, Robot-assisted surgery, Percutaneous endoscopic lumbar discectomy

## Abstract

**Background:**

To compare the clinical outcomes of patients with lumbar disc herniation treated with robot-assisted percutaneous endoscopic lumbar discectomy (r-PELD) or conventional PELD under fluoroscopy guidance (f-PELD).

**Methods:**

Our study group included 55 patients, 22 in the r-PELD group and 33 in the f-PELD group. The following clinical and surgical outcomes were compared between the two groups: the visual analog scale for radiculopathy pain; Oswestry Disability Index; intraoperative volume of blood loss; frequency of fluoroscopy used during the procedure; and MacNab classification. The follow-up period was 6–8 months.

**Results:**

Compared with f-PELD, r-PELD was associated with a lower volume of intraoperative blood loss and frequency of fluoroscopy (*p* < 0.01). There were no differences in complications, MacNab classification, postoperative disability and leg pain, and duration of hospitalization between the two groups.

**Conclusion:**

Based on our findings, r-PELD provides a safe and effective alternative to conventional PELD for the treatment of lumbar disc herniations, with the accuracy for placement of punctures lowering radiation exposure.

## Background

Conventional open discectomy has long been considered the standard treatment for lumbar disc herniation (LDH). However, complications and a lengthy recovery time remain the main drawbacks of this procedure. With recent advances in techniques for the treatment of LDH, percutaneous endoscopic lumbar discectomy (PELD) has become a widely accepted, minimally invasive treatment for LDH. PELD provides several advantages over open discectomy, including a shorter operative time and lower volume of blood loss [1, 2]. The main challenges of conventional PELD are radiation exposure and difficulty in performing the percutaneous puncture, especially in the high crista iliac and small foramen for a transforaminal route for L5-S1 LDH. Therefore, surgeons generally select to perform PELD at L5-S1 using an interlaminar approach, although radiation exposure during fluoroscopy-guided puncture is unavoidable.

In spinal surgery, the use of image-based guiding systems improves the efficiency and precision of the procedure [3]. It has been previously shown that navigation assistance, using a robot, can reduce operative time and provide better results than traditional radiography-guided surgery [4]. Our aim in this study was to compare clinical outcomes between robot-assisted PELD (r-PELD) and conventional fluoroscopy-assisted PELD (f-PELD) for the treatment of LDH.

## Methods

### Statement of ethics

The study was approved by our institute’s ethics committee. Patients provided informed consent for the treatment and use of their data for research and publication.

### Study group

This was a retrospective study. Eligible were patients who underwent PELD for LDH at our institution between April 2020 and October 2021. The inclusion criteria were as follows: unilateral radiculopathy caused by a single-level LDH and no meaningful improvement in symptoms after ≥ 3 months of conservative treatment. Excluded were patients whose symptoms were caused by spondylolisthesis, fracture, tuberculosis, or tumor; those with calcified LDH and lumbar canal stenosis; and those with a history of lumbar surgery. Of the 55 patients who met our selection criteria, 22 were treated with r-PELD (12 men; mean age, 39.2 years; age range, 15–59 years) and 33 with f-PELD (23 men; mean age, 43.4 years; age range, 19–71 years). 2 out of 22 patients underwent two levels surgery, while the rest of 22 patients underwent one levels surgery in r-PELD group. All of 33 patients underwent one levels surgery in r-PELD group.

### Surgical technique

**Fig. 1:**
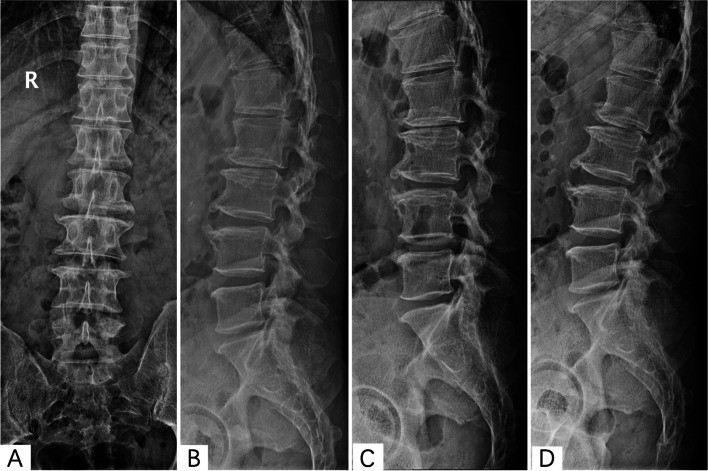
A 44-year-old male with L5/S1 lumbar disc herniation underwent PLED with Robot Assistance. **A** and **B**) Preoperative AP and lateral fluoroscopic images. **C** and **D**) Preoperative dynamic fluoroscopic images

**Fig. 2:**
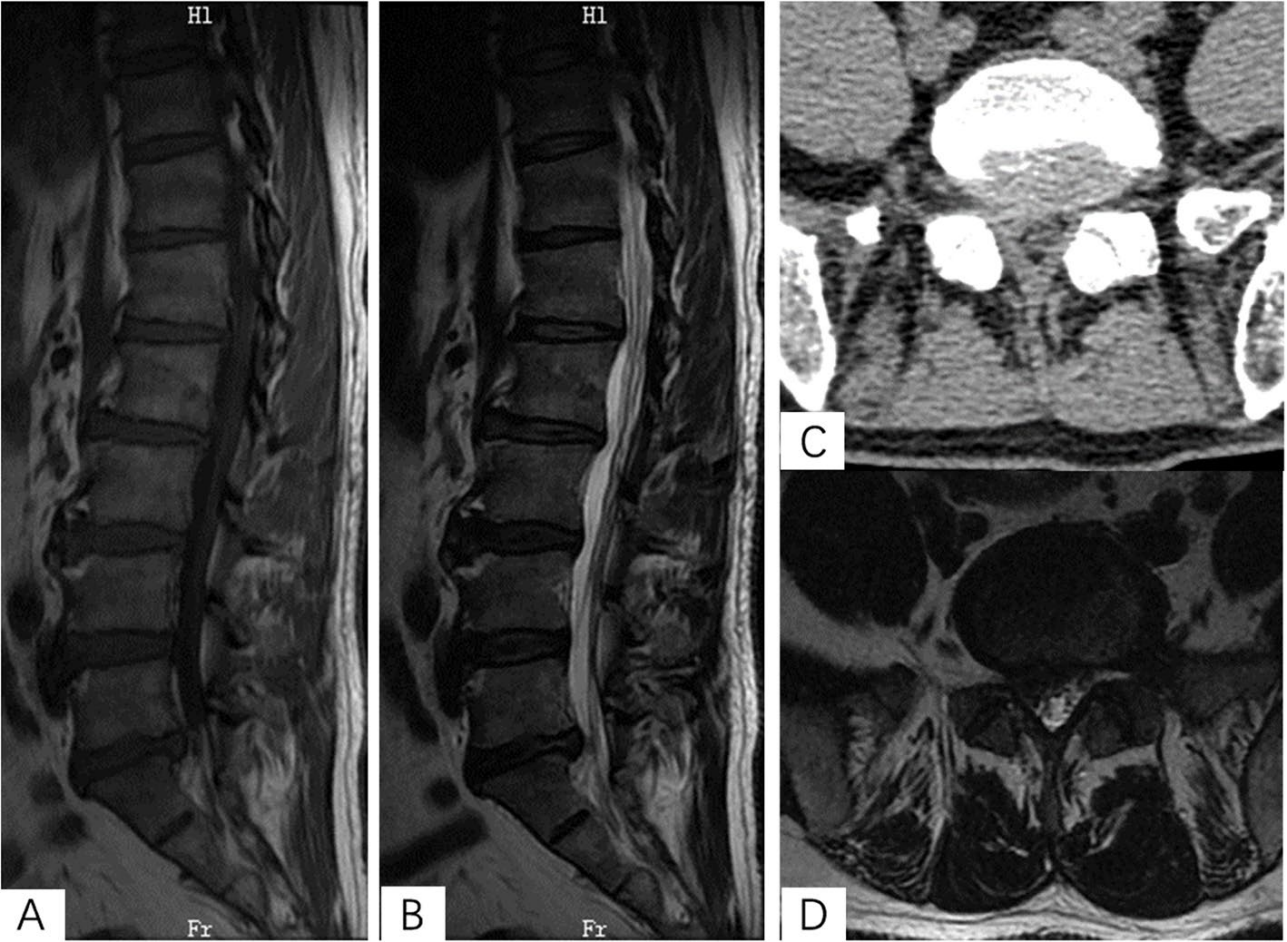
A 44-year-old male with L5/S1 lumbar disc herniation underwent PLED with Robot Assistance. **A**, **B** and **D**) Preoperative MRI images. **C**) Preoperative CT scan image

**Fig. 3:**
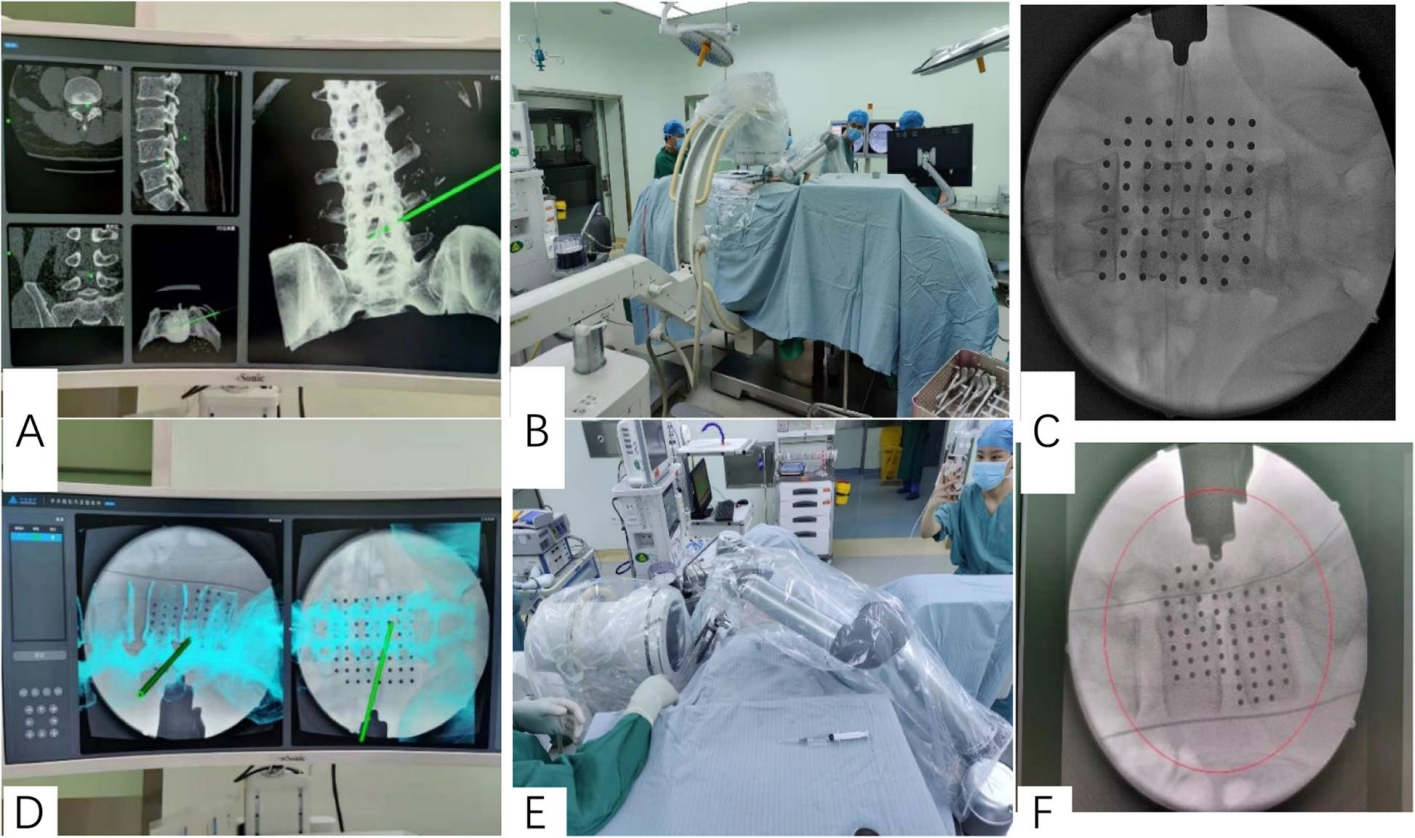
Fluoroscopic images are matched with CT data for the planning puncture trajectory. **A** The robotic system plans the puncture trajectory according to CT scan data is inputed into the working station. **B**, **C**, **E**, **F**) Anteroposterior and lateral fluoroscopic images are obtained while the robotic arm holds the reference frame. D)The fluoroscopic images are matched with the planning puncture trajectory based on CT data

The patients in the r-PELD group and f-PELD group were confirmed by computed tomography (CT), magnetic resonance (MR), and fluoroscopy imaging for the diagnosis of LDH (Figs. 1 and 2). Under local anesthetic, the patient was placed in the prone position on a radiolucent surgical table. C-arm and computer-assisted robotic system (Sanrobo robotic system; Santan Hangzhou, Zhejiang, China) were used to plan the puncture trajectory based on preoperative CT images (Fig. 3A). With the patient in position, anteroposterior (AP) and lateral images of the lumbar spine were obtained (Fig. 3B, C, E and F). The fluoroscopic images were matched to the planned puncture trajectory (Fig. 3D).

**Fig. 4:**
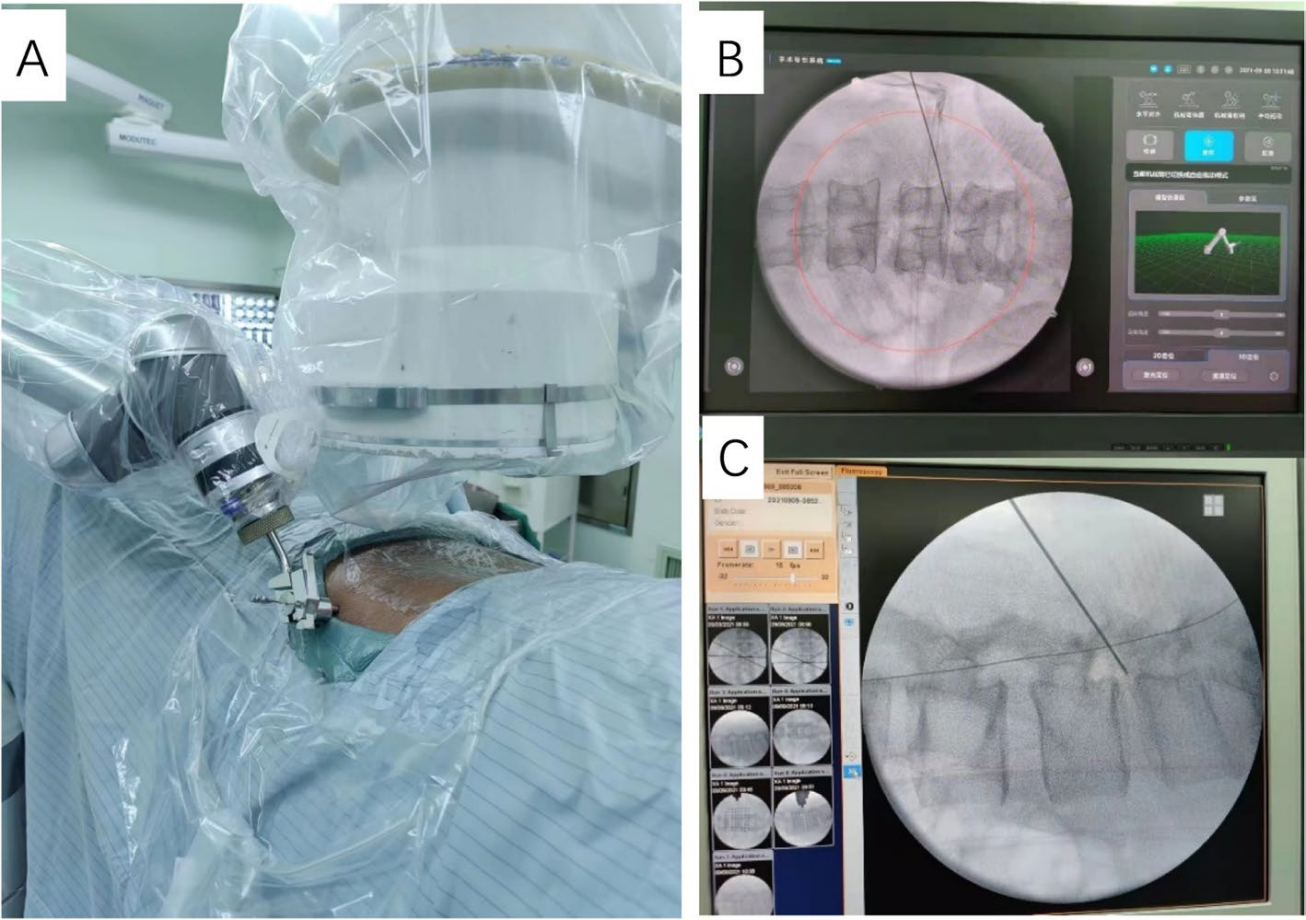
Process of puncture assisted by robotic system. **A** The robotic arm holds a spinal needle guided by the optimal trajectory for puncture after synthetizing image data. **B** and **C**) AP and lateral fluoroscopic images are obtained to confirm the position of the tip of the spinal needle after puncture

*In the r-PELD group*, punctures were performed as follows: the robot-assistance system synthesized imaging data and automatically reconstructed a 3D image of the foraminal zone, including a detailed analysis of the degree of stenosis, size and location of the herniated disc, and number of bony osteophytes. A realistic puncture trajectory was provided. For full decompression, substantially greater emphasis was placed on the facet joint during intraoperative facetoplasty. The robotic arm (Fig. 4A) held a spinal needle, guided by the optimal trajectory (from the tip of the superior facet joint to the AP part of the lower vertebrae body on lateral fluoroscopic image and the tip of the superior facet joint to the middle point of the inferior end plate of the target intervertebral space on the AP fluoroscopic image). Once the spinal needle progressed and docked on the facet joint, AP and lateral fluoroscopic images were obtained to confirm the position of the tip of the spinal needle (Fig. 4B, C). The working channel, endoscope, and instruments were then positioned directly onto the surface of the facet joint via a skin incision system with one-step dilatation. For the f-PELD group, punctures were guided by C-arm fluoroscopy as previously described [5, 6].

After successful insertion of the 6.3-mm endoscope, conventional facetoplasty and discectomy were performed in both groups.

### Statistical analysis

Continuous variables are presented as mean ± standard deviation (SD) and categorical variables as counts. Between-group comparisons were evaluated using the Mann–Whitney U test for continuous variables and the chi-square test for categorical variables, with a p-value < 0.05 considered statistically significant. All statistical analyses were performed using SPSS version 19.0 (IBM, Beijing, China).

## Results

The demographic data and LDH level are reported in Table 1.

**Table 1:** Demographic characteristics

	r-PELD	f-PELD	*p*-value
Number of patients	22	33	
Age	39.23 ± 11.86	43.46 ± 13.20	0.231464a
Sex			0.2702b
Male	12	23	
Female	10	10	
Height	171.23 ± 9.73	179.21 ± 8.66	0.686887
Weight	73.67 ± 14.98	70.25 ± 11.67	0.346079
BMI	24.85 ± 2.75	24.31 ± 3.29	0.525177
Involved level			
L1/2	0	0	
L2/3	2	2	
L3/4	0	2	
L4/5	14	10	
L5/S1	8	19	

### Clinical evaluation

Clinical data are summarized in Table 2. There were no between-group differences with regard to the duration of LDH before surgery, follow-up period, operative time, and days of hospitalization. However, the volume of intraoperative blood loss was lower in the r-PEDL group (12.14 ± 3.37 ml) than in the f-PELD group (15.55 ± 4.82 ml; *p* = 0.05) as was the frequency of intraoperative fluoroscopy (r-PELD, 2.91 ± 1.02; f-PELD, 9.94 ± 2.09; *p* < 0.01).

**Table 2:** Clinical data

	r-PELD	f-PELD	*p*-value	
**Disease duration (months)**	35.14 ± 33.47	40.47 ± 72.05	0.746187	
**Duration of operation (min)**	113.77 ± 49.70	120.70 ± 25.45	0.499614	
**Blood loss (ml)**	12.14 ± 3.37	15.55 ± 4.82	0.005784	
**Follow-up time (months)**	6.18 ± 2.46	7.73 ± 4.09	0.118019	
**Frequency of intra-operative fluoroscopy**	2.91 ± 1.02	9.94 ± 2.09	< 0.000001	
**Hospitalization days**	5.05 ± 1.00	5.42 ± 2.17	0.446997	
**VAS for leg pain**				
Pre-OR	5.64 ± 1.33	5.30 ± 1.08	0.310255	
Post-OR Immediately	0.64 ± 0.66	0.58 ± 0.66	0.740329	
Post-OR 3 days	0.68 ± 0.48	0.46 ± 0.56	0.126021	
Post-OR 1 month	0.09 ± 0.29	0.12 ± 0.42	0.768403	
**ODI**				
Pre-OR	29.14 ± 3.73	27 ± 5.79	0.132045	
Post-OR Immediately	6.77 ± 1.80	6.18 ± 1.31	0.164214	
Post-OR 3 days	6.09 ± 1.44	6.21 ± 1.47	0.764474	
Post-OR 1 month	3.23 ± 1.07	3.70 ± 1.83	0.282335	
**Complications**				
Nerve root injury	0	0		
Nerve root sleeves rupture	0	0		
Dural sac rupture or leakage of CSF	0	0		
Infection	0	0		
Hematoma	0	0		
Increased weakness of quadriceps or foot/toe extensor strength	6	3	0.1340	
Rebound of leg pain	0	0		
Residual or recurrence	0	0		

The VAS score for leg pain and the ODI are reported in Table 2 and did not differ between the two groups. Similarly, there was no between-group difference in the MacNab classification (Table 3), with a classification score of 100% for the r-PELD group and 98% for the f-PELD group. The rate of complications was also not different between the groups (*p* = 0.13; Table 2) and included three cases of quadriceps or foot/toe extensor weakness in the f-PELD group; there were no cases of infection, hematoma, dural rupture, or nerve root injury.

**Table 3:** MacNab classification data after surgery

	r-PELD	f-PELD	
**MacNab classification data**			
Excellent	18	26	
Good	4	6	
Fair	0	1	
Poor	0	0	
Excellent or good rate	100%	97%	
*p*-value^a^	> 0.9999		

## Discussion

PELD is increasingly used for the treatment of patients with radiculopathy caused by disc herniation, with satisfactory results achieved and lowering the risk of complications. Compared to traditional open surgery or microendoscopic discectomy, the working channel, endoscope, and instruments for PELD are placed directly on the surface of the lamina or facet joint through a 0.7-cm skin incision during a one-step dilatation. The use of a smaller incision and direct implantation of the working cannula, without the need for muscle dissection, may improve postoperative recovery. As a result, one of the benefits of PELD is a lower risk of approach-related morbidity. Intraoperative bleeding is also effectively decreased through the use of a continuous fluid flow or irrigation system, as well as the use of an endoscopic-specific radio frequency and bipolar coagulation. In most cases, bone resection, performed using a small diamond burr via transforaminal technique or an interlaminar approach, is required to achieve a full recovery.

Since the 1990s, robots have been used in spinal surgery, in particular for pedicle screw implantation, percutaneous vertebroplasty, spinal biopsy, and tumor resection, providing real-time intraoperative navigation for increased accuracy while lowering radiation exposure [4, 7].While traditional PELD uses C-arm fluoroscopy for guidance, exposing patients, surgeons, and surgical personnel to high levels of radiation and increasing procedural time, our robot-assisted procedure uses C-arm fluoroscopy to obtain initial AP and lateral images for guidance, lowering the radiation exposure by reducing the frequency of intraoperative fluoroscopy (*p* < 0.01). More recently, a new generation of robot assistance systems have been developed, which provide 3D anatomical imaging of the spine based on synthesis of AP and lateral view images of the lumbar spine, obtained intraoperatively or on preoperative CT imaging, without the need for a fixed dynamic frame. We do note that change in the position of patients placed in the lateral decubitus position on the operative table can lead to significant navigation system errors. To avoid such errors, we placed our patients in the prone position. However, in our first two patients, we noted that they were in the supine position for preoperative CT imaging and for intraoperative initial AP and lateral imaging. This difference, with the prone intraoperative position during the procedure, caused a mismatch in the navigational 3D anatomical images, which led to difficulty or mistakes in the path of puncturing during PELD. To correct for this error, we placed patients in the prone position for preoperative CT images, which increased the accuracy of puncture and lowered radiation exposure.

Less experienced surgeons may require a longer time for a puncture, resulting in larger radiation doses due to the high learning curve for the PELD puncture procedure [8, 9]. Higher radiation doses may also occur for experienced surgeons performing PELD at the L5/S1 level in patients with a high iliac crest. Considering the negative health effects of cumulative radiation exposure, there is an urgent need for spinal surgeons to adopt image-guided systems. Ultrasound-guided PELD has been shown to reduce radiation exposure while providing real-time guidance [10]. However, the hyperechoic zone in the L5-S1 region can limit the required visibility for a puncture at this level, with this issue being particularly problematic for patients with a high iliac crest [11]. The robot assistance system we used in our study provided created a satisfactory trajectory at L5-S1 with a high iliac crest, with two fluoroscopy images required, on average, per procedure. In our study, the higher rate of puncture accuracy in the r-PELD group minimized the invasiveness of the procedure, with a lower volume of blood loss and more effective removal of the superior articular process (SAP) for the r-PELD group than that for the f-PELD group. The SAP limits the manipulated space available to position the working channel by obstructing access to the anterior epidural region. Through full decompression of the ventral and dorsal structures by enlargement of the foramen by undercutting of the ventral section of the SAP and eliminating the foraminal ligament, foraminoplasty that eliminates part of the SAP can enable the direct vision of the anterior epidural space [12]. Transforminal PELD performed with foraminoplasty, when it was safe to do so, resulted in a 92.5% patient satisfaction rate [13]. The reported failure rate of PELD without foraminoplasty ranges from 4.3–10.3% [14, 15]. In our study, compared to conventional PELD guided by fluoroscopy, robot assistance provided a visible trajectory for puncture to the targeted protruding nucleus pulposus, with minimal SAP bone removal, maintaining the anatomical structure of the SAP to the extent possible for lumbar stability and significantly reducing the duration and difficulty of puncture and the rate of nerve root injury (especially for elderly patients with hypertrophic facet joint and lateral recess stenosis).

For most spinal surgeries, image guiding systems allow surgeons to achieve higher precision and efficiency [16].Surgical tools can be tracked in three-dimensional (3D) space during spinal surgery using image-based navigation, allowing surgeons to navigate the spinal anatomy using pre- or intraoperative CT imaging data. Intraoperative navigation for spinal decompression and fixation improves surgeons’ ability to visualize the structures and neurovascular systems of the spine, supporting more effective and faster procedures, as well as shortening operative time and improving the accuracy of screw placement accuracy for adequate decompression [16–18]. One of the distinct benefits of guided techniques is the visible trajectory for insertion of the dilator and working channel at the surgical level, with a suitable incision site, including for patients for whom localization information is difficult to obtain by radiography [19].

Previous studies have established that percutaneous transforaminal endoscopic discectomy can achieve adequate decompression for different types of LDH, including central, paracentral, foraminal, extraforaminal, and calcified LDH [20, 21], which are dependent on the location of the protruding nucleus pulposus. A previous study reported an association between a failure of decompression and the location of the protruding nucleus pulposus [22]. Considering that nerve compression is the core symptom guiding PELD [20], establishing an ideal working cannula to reach the lesion is a key outcome of the PELD procedure [23].

Relying on a reference frame fixed to the bone or surgical table, as well as a large distance between the navigation instruments and the reference frame, can cause navigation errors when using O-arm-based systems [24, 25]. The robot assistance system used in our study relies on a combination of preoperative CT scans and intraoperative fluoroscopy images. By placing patients in the same prone position for preoperative CT and intraoperative imaging, we were able to eliminate navigational error using the r-PELD procedure. However, further research is warranted to fully clarify the accuracy of the robot-assist system we used, particularly for LDH treatment at the L5-S1 level. We also note the higher cost for robot-assisted surgery in our study, compared to conventional f-PELD, with the cost borne by patients, as well as the time-consuming nature of processing the preoperative CT and intraoperative fluoroscopy imaging data, which may make patients uncomfortable without any anesthesia. This processing time might account for the lack of difference in the duration of procedure between the two groups despite robot assistance for puncture.

## Conclusion

For the treatment of LDH, robot assistance systems are safe, effective, and less invasive than conventional f-PELD. The robot system used provided real-time guidance to facilitate the placement of punctures.

## Data Availability

The data presented in this study are available in the article.

## Abbreviations

CT: Computed tomography
LDH: Lumbar disc herniation
MICEPS: Minimally invasive cervical pedicle screw
MR: Magnetic resonance
PELD: Percutaneous endoscopic lumbar discectomy
SAP: Superior articular process
SD: Standard deviation
